# Seiðr: Efficient calculation of robust ensemble gene networks

**DOI:** 10.1016/j.heliyon.2023.e16811

**Published:** 2023-05-31

**Authors:** Bastian Schiffthaler, Elena van Zalen, Alonso R. Serrano, Nathaniel R. Street, Nicolas Delhomme

**Affiliations:** a Department of Plant Physiology, Umea Plant Science Center, Umea University, Umea, Sweden; b Department of Plant Physiology, Umea Plant Science Center, Swedish University of Agricultural Sciences, Umea, Sweden

**Keywords:** Gene network inference, Gene regulatory network, Gene co-expression network, Systems biology, Functional genomics

## Abstract

Gene regulatory and gene co-expression networks are powerful research tools for identifying biological signal within high-dimensional gene expression data. In recent years, research has focused on addressing shortcomings of these techniques with regard to the low signal-to-noise ratio, non-linear interactions and dataset dependent biases of published methods. Furthermore, it has been shown that aggregating networks from multiple methods provides improved results. Despite this, few useable and scalable software tools have been implemented to perform such best-practice analyses. Here, we present Seidr (stylized Seiðr), a software toolkit designed to assist scientists in gene regulatory and gene co-expression network inference. Seidr creates community networks to reduce algorithmic bias and utilizes noise corrected network backboning to prune noisy edges in the networks.

Using benchmarks in real-world conditions across three eukaryotic model organisms, *Saccharomyces cerevisiae*, *Drosophila melanogaster*, and *Arabidopsis thaliana*, we show that individual algorithms are biased toward functional evidence for certain gene-gene interactions. We further demonstrate that the community network is less biased, providing robust performance across different standards and comparisons for the model organisms.

Finally, we apply Seidr to a network of drought stress in Norway spruce (Picea abies (L.) H. Krast) as an example application in a non-model species. We demonstrate the use of a network inferred using Seidr for identifying key components, communities and suggesting gene function for non-annotated genes.

## Key points

• Seidr is the first native implementation of the Marbach et al. paradigm: an ensemble of inference outperforms any predictor.
• Seidr independently validates Marbach's paradigm.
• Seidr addresses the computational limitations of the most common inference methods, making them applicable to large datasets.
• Seidr is applicable in both model and non-model organisms.
• Seidr helps to unravel novel biology and to transfer gene annotation to genes of unknown function.

## 1 Introduction

The increasing accessibility of RNA sequencing in recent years has popularized large scale applications of computational biology. Two such methods, gene regulatory network (GRN) and gene co-expression network (GCN) inference, derive network structures of either transcription factor (TF) to target, or generic gene-gene associations, from gene expression data. The primary goal of a GRN studies is the discovery of new regulatory interactions of known transcription factors, often in specific conditions or during certain developmental stages, or the identification of high-impact candidate genes for biotechnology applications and genome engineering [1,2]. Conversely, GCN analysis assists researchers in functional annotation and gene-phenotype association studies [3].

The area of computational inference of GRNs and GCNs receives considerable attention from the scientific community with new software published regularly, but the challenging nature of the problem makes progress in the field incremental. Gene networks tend to suffer from two main issues: inference algorithm s are often biased toward (or against) some types of regulatory interactions [4] while the experimental limitations, such as the number of samples and precision of sampling, often lead to low signal-to-noise ratios. To address the first bias, Marbach et al. [4] proposed a voting-based scheme of a “crowd” of networks (hereinafter referred to as a community), which increased the robustness of the final aggregate network both on simulated and real data. To counteract the low signal-to-noise ratio limitation, Coscia and Neffke recently proposed a network “backboning” strategy that employs a Bayesian framework to filter non-essential edges from a dense network [5].

Despite the se proposed methods few studies in the field make use of either of them. On one hand, published methods often rely on a single inference method and a naïve edge threshold selection, where all edges below an arbitrary cutoff are filtered. On the other hand, the few software implementation of these methods that exist [4,6,7] are proof-of-concepts that are limited in their applicability and scalability due to the integration of unoptimized code, hence unsuitable for high-throughput analysis. These caveats severely limits the scale of the networks that can be inferred, typically focusing on a limited number of edges, allowing only for the inference of interactions between known transcription factors and putative target genes as opposed to more comprehensive all-vs-all analyses.

We have developed “Seidr” (stylized “Seiðr”), a feature rich software package that currently implements ensemble network inference, aggregation, backboning as well as numerous tools to interact with Seidr networks. An important semantic distinction should be made between our approach to group into an ensemble a set of different inferences methods, i.e. building a meta-network of inference networks, and single individual inference algorithms that relies on an ensemble learning method, such as EnGrain [53] and EnGRNT [54]. In other words, Seidr could be used to aggregate such ensemble learning methods’ inferences into the meta-network it generates.

Seidr is written in C++, can scale to tens of thousands of genes and can be executed on high performance compute clusters using message parsing interface (MPI) distributed computing, making it applicable for high-dimensional analyses, including large single-cell datasets.

In a nutshell, the novelty of Seidr is its comprehensive, efficient, and robust integration of existing network inference approaches (including their faster re-implementation whenever needed), its ability to aggregate their results into an ensemble, to subsequently retain the informative edges, and to allow for network metrics and analysis, *e.g.* partitioning, to be conducted thanks to the integration of the most relevant methods available, often, from other scientific fields. It comes with its own file format (sf), inspired by the Sequence Alignment Map (SAM) format [55], which is both storage and computationally efficient and allows for the afore-mentioned steps to be natively performed.

We show that Seidr produces robust results in three networks produced from real-world bulk RNA sequencing data of three model eukaryotic species, aggregating the results of 13 inference methods on datasets consisting of over a thousand samples. We chose to utilize real-world data as benchmarks for GRN and GCN inference software, as *in silico* gold standards are not always reliable predictors for performance on real world data, due to them being inherently subject to assumptions about the dynamics between expression quantification and gene interaction, or relying only on evidence from simpler prokaryotic species.

Finally, to demonstrate a typical use-case, we apply Seidr to a study of drought stress in Norway spruce (Picea abies (L.) H. Krast).

## 2 Results

### 2.1 Seidr workflow

Currently, thirteen gene network inference methods are implemented in Seidr, in three broad inference groups: correlation, mututal information (MI), and regression. Correlation based methods include Pearson correlation, Spearman correlation, the topological overlap metric (TOM) from WGCNA [8] and a shrinkage estimate to partial correlation (PCOR) as implemented in Schäfer and Strimmer [9]. In the MI group Seidr supports calculating raw MI scores using B-splines [10], which can be further post-processed using the CLR [11], or ARACNE [12] algorithms. The fourth MI method implements NARROMI [13]. In the regression group, Seidr provides GENIE3 [14], TIGRESS [15], linear SVM (LLR) and Elastic Net ensembles (ELNET) [16], and PLSNET [17].

**Fig. 1:**
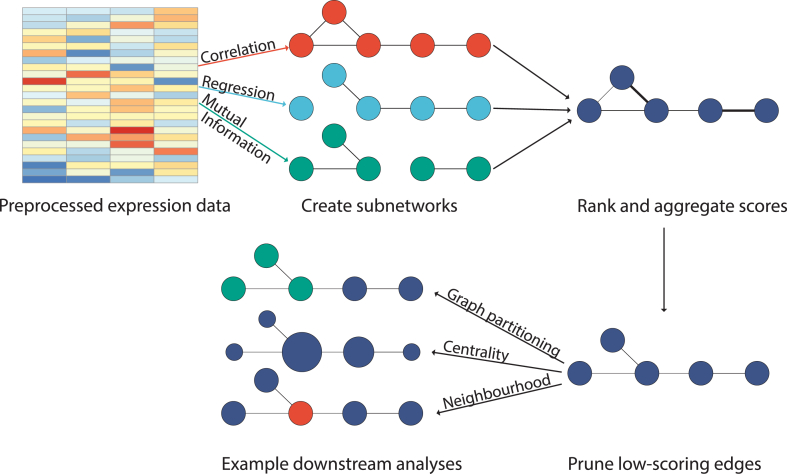
Typical Seidr pipeline. Pre-processed expression data is input into any number of network inference programs, e. g., those that are implemented in Seidr itself. These networks are then rank-aggregated into a community network using a supported aggregation scheme. Networks can optionally be pruned via noise-corrected dynamic backboning or naïvely by a score cutoff. Finally, numerous downstream procedures can be used to analyse the resulting networks.

A typical Seidr workflow involves three processing steps (a representation can be seen in Fig. 1), which are further detailed in the following three sections, before the resulting filtered ensemble-network can be mined for biological insights, as we will demonstrate further on, through an application on Norway spruce.

1. **Inference**: Construct any number of independent gene networks using any combination of the afore-mentioned algorithms. This can be done for a full all-vs-all gene network or in a gene set targeted approach (*e.g.*, only transcription factors).
2. **Aggregation**: The group of inferred networks is aggregated into a community network using one of the supported aggregation methods.
3. **Filtering**: As aggregation usually outputs a fully connected network, it is desirable to cut low confidence edges. Seidr can be used to estimate a hard threshold, using network scale-free fit and transitivity as lead statistics, or by applying a dynamic cutoff as suggested in Coscia and Neffke [5].
4. D**ownstream analysis**: A dense or pruned network can then be utilized in a number of graph-based analyses. Common choices include graph partitioning, centrality analysis or neighborhood analyses in order to study genes (or groups of genes) related to a function of interest.

In the following, we will first evaluate the effect of arbitrary *vs.* dynamic edge trimming, then report on our assessment between individual *vs.* community networks, prior to demonstrating downstream analysis conducted in Norway spruce on a drought stress dataset.

### 2.2 Benchmark data and gold standards

To benchmark the performance of algorithms on real-world data we selected publicly available RNA-Seq datasets and retrieved three independent resources as gold-standards. The data was obtained from the National Center for Biotechnology Information (NCBI) Sequence Read Archive (SRA) for three model species: *S. cerevisiae* (N = 1399), *D. melanogaster* (N = 859) and *A. thaliana* (N = 1216) and consisted of a mix of experiments, some done at a steady state (*e.g.,* mutant *vs.* control) and some originating from time-series studies. As for the standards, we selected the Biological General Repository for Interaction Datasets (BioGRID) interaction dataset for each species as a measure of ground truth for general connectivity [18]. For functional proximity, we used the Kyoto Encyclopedia of Genes and Genomes (KEGG) database as a proxy, defining an edge between two genes if they co-occur in a pathway [19]. For *S. cerevisiae*, we chose the Yeast Search for Transcriptional Regulators And Consensus Tracking (YEASTRACT) database as a measure of regulatory proximity [20]. In order to verify the predicted networks, we assessed whether individual or community methods predicted higher-scoring edges for in-groups (inferred edge present in the standard) compared to out-groups (predicted edge absent from the standard). For KEGG, we tested the following null hypothesis: links between genes annotated to the same pathway do not receive higher weights compared to genes annotated to different pathways. For YEASTRACT and BioGRID, we formulated the null hypothesis as follows: links present in the gold standard - treated as undirected - do not receive higher weights compared to links of the same genes to those outside of the gold standard. As can be seen in Supplementary document S2 and Supplementary Figures S1 to S7, all inference methods consistently predicted significantly higher scoring edges for the ingroups over the outgroups (one-sided Kruskal-Wallis tests). These strong associations observed for all single methods were on par with those from the Seidr community. Note that an assessment of the Seidr community performance over that of the individual networks in that analysis is irrelevant as the quality (sparse vs. dense) of the networks is not controlled for. This will be addressed further below. For now, we have independently re-asserted that all methods, whether single or an aggregate perform as expected and that all the 13 single inferences methods are worth aggregating into a Seidr community, irrespectively of the organism or the gold standard used. This suggests a global applicability of the approach, at least for eukaryotes.

### 2.3 Network pruning improves sensitivity and specificity

Many gene network inference algorithms produce dense networks, meaning every gene is connected to every other gene with a calculated edge weight. To prune these networks, researchers often perform naïve pruning, which cuts all edges below an arbitrary threshold. Coscia and Neffke [5] argued that this method fails to properly take local edge distributions into account, and therefore fails to separate signal from noise accurately. We implemented their algorithm in Seidr and performed backboning for all δ (the standard deviation of the expected variance of edge weights connected to a single node) in range [0.1,0.2,...,3.5] for all algorithms implemented in Seidr and all benchmark networks. We further generated naïvely pruned networks with approximately equal edge counts to their backbone filtered counterparts. We then used all standards to generate receiver operator characteristic (ROC) and precision-recall (PR) curves for all aforementioned combinations and calculated the area under the ROC-curve (AUC) and under the PR-curve (AUPR). Briefly, the AUC reports whether a predictor ranks true positives higher than false positives and the AUPR shows how relevant the edges it highly ranks are. An AUC=1 indicates a perfect predictor and AUC=0.5 indicates a random predictor. We only considered networks with greater than 0.1% edge density (*i.e.,* the fraction of edges left in the network compared to all theoretical edges if all genes were connected to each other), as otherwise the number of gold standard edges would be too sparse to generate ROC curves.

In general, all networks benefited from filtering edges using either method. Most networks improved by lenient backboning, pruning edges below a δ of 2, at which the benefit reached a plateau (see Supplementary document S2 and Supplementary Figure 8 and 9). Pruning stricter than a δ of 2.32 - which represents an approximate *P*-value of 1% - often resulted in a decrease in AUPR and a small increase in AUC, representing a trade off between sensitivity and specificity. It is worth to note that in this analysis both the lenient and hard filtering seem to perform equally well, something that is probably compounded by the fact that we selected a hard threshold using a similar number of edges than present in the backbone, i.e., we took advantage of an information (the number of edges) that would not have been available to us had we based our hard threshold cutoff solely on measures such as the scale-free transition (SFT that maximizes the R2 of the scale free distribution) or the average clustering coefficient (ACC).

### 2.4 Ensembles of networks improve robustness

**Fig. 2:**
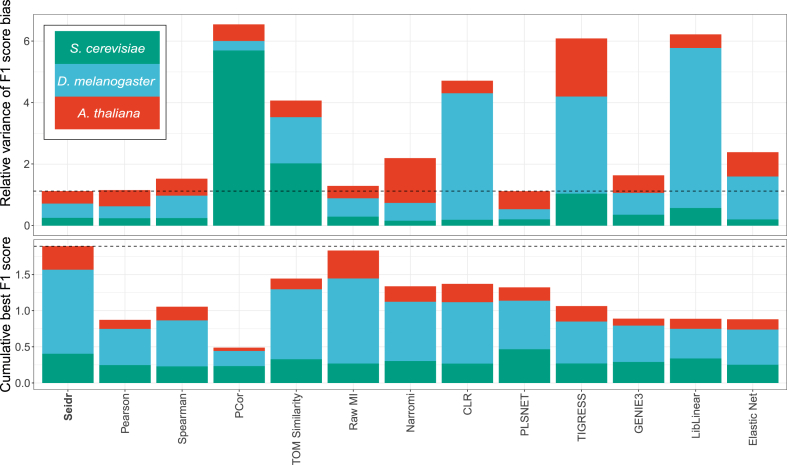
**Top:** Estimate of method bias, lower is better. Networks were evaluated on standards derived from single BioGRID evidence types and compared to all edges from BioGRID. Increased or decreased evaluation scores (F1) suggests bias of the method toward a specific type of evidence. This plot shows the relative variance of F1 scores from subsets compared to the full dataset. **Bottom:** Cumulative method performance across all evaluations, F1 scores. For all three species as well as KEGG, BioGRID and YEASTRACT standard, the highest performing pruned network was selected for each method. The bar plot shows the sum of maximal F1 scores, shaded by species.

Marbach et al. [4] suggested that community networks are superior to any single method due to their resilience against algorithmic bias. This idea was further tested using phosphorylation data by Hill et al. [21], who showed that community approaches can benefit causal networks. To understand whether different gene network inference algorithms are biased toward certain types of interactions, we created subsets of the full BioGRID standard. We split the dataset by the type of evidence as a proxy for the interaction type. Next, we created a community network for each benchmark dataset, using the inverse rank product (IRP) algorithm [22], and performed the same pruning steps as for all other networks. We then recomputed the F1 scores (a measure of accuracy that combines precision and sensitivity) from the AUC and AUPR solely based on each single evidence group and compared the mean F1 of the evidence subset to the mean F1 of the full dataset. We summarized general robustness as the relative variance of mean differences to the baseline (Fig. 2, top), which ranks the community network as the best cumulative score, about even with raw MI.

In order to understand whether the community network performs better than any single method, we aggregated all benchmark data and selected the highest scoring pruned network for each method. The sum of performance evaluations over all standards and species ranked Seidr as the highest scoring method, followed by raw MI (Fig. 2, bottom), independently validating Marbach's paradigm in all vs. all gene network inferences of large datasets across several eukaryote model organisms. Having demonstrated the relevance and applicability of Seidr, we moved on to apply it to a non-model organism: Norway spruce, and demonstrate its usefulness for data-driven, hypothesis generating biological studies by identifying changes induced in the network by an abiotic stress and highlighting candidate genes for downstream hypothesis driven validation studies.

### 2.5 Applying seidr to drought stress in Norway spruce

#### 2.5.1 Drought and non-drought networks

In order to identify candidate drought-specific genes in Norway spruce, we used Seidr to create a network of drought stressed needles using previously published RNA-Sequencing data [23]. Briefly, in this experiment the authors assayed physiological and transcriptional changes in Norway spruce seedlings after inducing drought stress. Within the first five days, soil moisture was reduced from 80% field capacity (FC) to 30% FC and subsequently held at 30% for seven days. Severe drought stress was then induced by withholding water until severe symptoms of dysfunction occurred. These conditions were held for four days after which re-irrigation started. Four days after re-irrigation started, soil moisture had returned to 80% FC. Needles were sampled at control (day zero), mild (two, four, five and 13 days) and severe (18 and 21 days) drought and after re-irrigation (25 days).

After calculating a gene network from this data, we further performed edge pruning via the backbone method, graph partitioning using InfoMap [24] and calculated node-based centrality statistics, all steps using convenience functions implemented in Seidr. In parallel, we followed the same pipeline using a compendium of RNA-Seq data from unstressed needles as a non-specific dataset in order to compare both networks, assess whether their structure differed in terms of node and edge statistics, and communities, before focusing on a subset of novel candidate genes.

#### 2.5.2 Higher node centrality coincides with relevant biological functions

To investigate drought-related gene functions within the gene networks, we first used a curated list of Norway spruce orthologs of *Arabidopsis thaliana* genes with confirmed roles in drought stress [23] (N = 150, see supplementary data S3) and used Seidr-calculated node centrality metrics for each node in the stressed and unstressed networks. We then tested whether genes in our curated dataset had significantly higher centrality values compared to all other genes or compared to a random sample of genes of the same magnitude (one-sided Kruskal-Wallis, FDR: $\alpha <2.8*10^{-4}$). The set of curated genes was significantly higher ranked in all six centrality statistics only in the drought-specific network. While the same nodes also showed a similar trend in the non-specific network, none of the associations were statistically significant (Supplementary document S2 and Supplementary Figures S10-12). In addition, we used gene set enrichment analysis (GSEA) to test if the set of curated genes was enriched for high-centrality nodes in either network. Within the stressed network, all metrics showed a high degree of association between centrality and the set of curated genes, whereas the unstressed network only had significant enrichment in one out of six statistics (betweenness, Supplementary document S2 and Supplementary Figures S10-12).

To supplement the previous analysis with unbiased resources, we performed gene set enrichment analysis of gene ontology (GO) categories for both networks using the node centrality values as covariates (supplementary data S4). Nodes with high centrality in the stressed network were enriched for numerous response processes to biotic and abiotic stimuli, as well as metabolic processes such as nicotinamide adenine dinucleotide phosphate (NADPH) regeneration (GO:0006740), lipid biosynthesis (GO:0008610) and the mitogen-activated protein kinase (MAPK) cascade (GO:0000165). In contrast, nodes with high centrality in the unstressed network were enriched for processes such as developmental growth (GO; 0048,589), cell cycle (GO:0007049) and cytokinesis (GO:0000910).

These results asserted that biologically relevant nodes can be identified by reconstructing networks from datasets aimed at studying specific gene expression perturbations.

#### 2.5.3 Partitioning and enrichment reveal communities with relevant biological functions

**Fig. 3:**
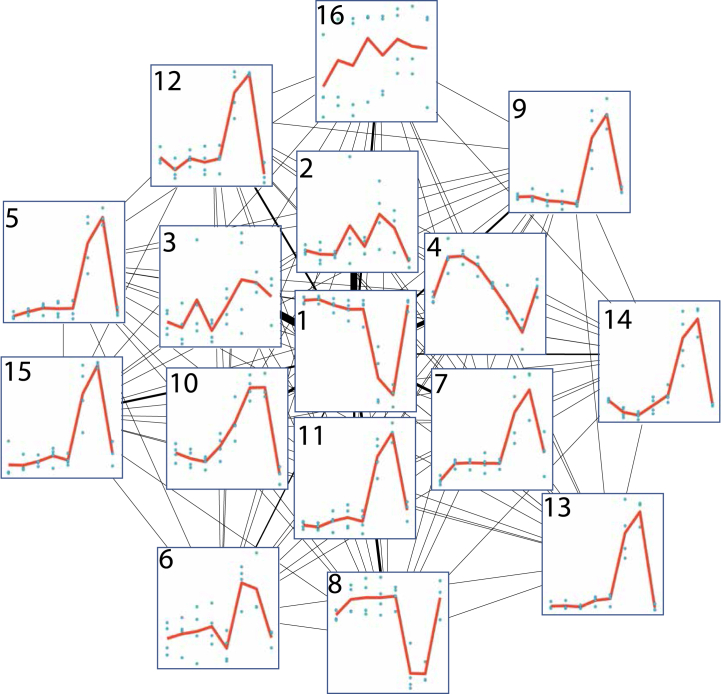
Network connectivity plot of network modules in an experiment tracking drought stress in Picea abies. The module number and eigengene (defined as the first principal component of the variance stabilized expression) are shown as each node, connections between nodes are represented as edges, where edge width highlights the strength of the information flow. The x-axis represents the soil hydration, with the following eight successive steps: 80%, 60%, 40%, 30%, after seven days of continuous drought at 30% hydration, collapse in function of photosynthesis and transpiration (indicating severe dysfunction), continued collapse after two days and finally re-hydration to 80%.

Using the edge-pruned drought network, we partitioned the graph via InfoMap [24] (Fig. 3). For each top-level module in the graph partition with more than 10 member genes (N = 16), we performed gene enrichment analysis using the GO [25,26] (supplementary data S5) and MapMan [27] (supplementary data S6) databases as annotation. Modules 2, 5 and 6 al l were significantly enriched for stress response terms ($P_{adj}<0.01$). Module 2 was enriched for “defense response” (GO:0006952, $P_{adj}=2.82e^{-11}$) and “response to stimulus” (GO:0050,896, $P_{adj}=2.87e^{-3}$). Module 5 was enriched for “response to oxidative stress” (GO:0006979, $P_{adj}=2.27e^{-6}$), “response to chemical” (GO:0042,221, $P_{adj}=1.62e^{-4}$), and “response to redox state” (GO:0051,775, $P_{adj}=5.2e^{-4}$). Finally, module 6 was enriched for “response to karrikin” - a group of plant hormones found in the smoke of burning plant material (GO:0080,167, $P_{adj}=1.28e^{-6}$).

These results further supported the structural validity of the reconstructed network as well as the applicability of Seidr to non-model organisms, comforting us into mining the network for novel drought-relevant candidate genes to be used in future validation studies.

#### 2.5.4 High-centrality transcription factor analysis reveals novel Norway spruce specific drought-response candidate genes

**Fig. 4:**
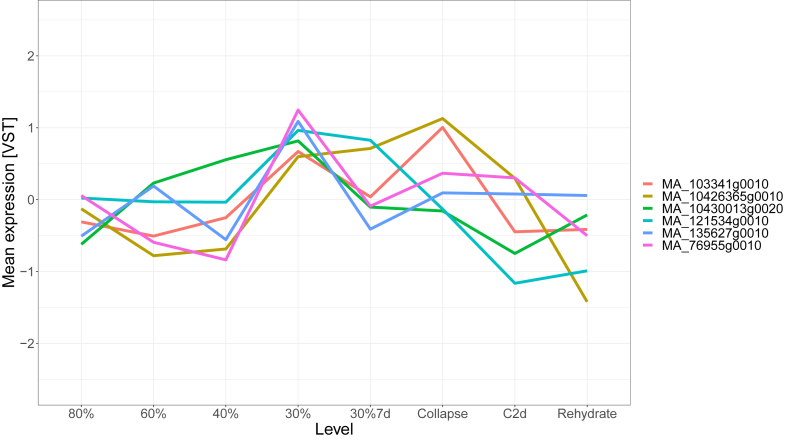
Mean expression of high-centrality transcription factors identified in module 2. Expression values are variance stabilized counts, biological replicates were summarized by their mean value. The x-axis represents the soil hydration. Data points “30%7 d”, “Collapse”, “C2d” and “Rehydrate” represent seven days of continuous drought at 30% hydration, collapse in function of photosynthesis and transpiration (indicating severe dysfunction), continued collapse after two days and finally re-hydration to 80%.

In order to find possible targets for genetic modification, we calculated median-rank centrality values for all annotated transcription factors (TFs) in the network and selected the top 20 nodes. We then further narrowed down the list by only selecting transcription factors in modules 2, 5 or 6 given the previous analysis related to GO enrichment in these modules. None of the TFs were members of modules 5 (response to oxidative stress, chemicals, or redox state) and 6 (response to Karrikin), but a total of six (30%) were annotated to module 2. All of these show a characteristic expression peak at 30% hydration, with “MA_103341g0010” (a NAC - NAM, ATAF1/2 and CUC2 - domain-containing 35-like TF) and “MA_10426365g0010” (an NAC domain-containing 86-like TF) peaking again during plant collapse (Fig. 4). None of the TFs in module 2 were present in the curated list of genes involved in drought response. This is unsurprising, given the facts that Norway spruce annotations have all been lifted in-silico from the plant model *Arabidopsis thaliana* and the evident genetic differences between the two species, and on the contrary very promising about insights into spruce specific physiology. While the subject of a follow up study, it also suggests the possibility to use the network's structure to infer functional information for unannotated genes based on their surrounding community.

#### 2.5.5 Local neighborhood as a mean of functional annotation inference

In order to assess functional inference power in the stressed network we selected three non-annotated genes with high median-rank centrality. In their local neighborhood we then selected nodes directly connected to the non-annotated gene.

Gene **MA_10432675g0010** contains a cellulase (glycosyl hydrolase family 5) domain. Local neighborhood connects it to MA_10425958g0010 (Kinesin, mitochondrial isoform ×1), MA_4391326g0010 (another cellulase) and MA_84105g0010, a glycoside hydrolase. Cellulases are part of the cell wall remodeling mechanisms triggered by abiotic stresses.

Gene **MA_69177g0010** has two non-annotated neighbours - MA_136426g0010 and MA_894367g0010 - as well as MA_41206g0010 which is annotated as a Mitogen-Activated Protein Kinase 3. MAPK are involved in signal transduction, from a diverse array of stimuli, including osmotic stress.

Gene **MA_10192193g0010**. This gene has two neighbours. MA_10192193g0020, annotated as zinc finger CONSTANS-LIKE 16, and MA_10226519g0010, which is a DNA photolyase. CONSTANS is involved in photoperiod sensing in *Arabidopsis thaliana*, and photosynthetic changes are induced in plants by drought stress.

While these three examples remain superficial, they are strongly indicative of the potential that lies in the network structure for the selection of candidate genes, as well as for functional annotation inference.

## 3 Discussion

Gene network inference, be it regulatory or co-expression networks, is an important and widely used bioinformatics tool for modern biological research, but best practice information and implementations are lacking. This consideration is especially relevant now, as the single cell sequencing boom is generating increasingly massive-scale expression datasets that synergize well with gene network analysis given their high sample count [28]. However, care must be taken as to which methods are selected to perform the inference [29], with several new methods proposed to specifically address network inference in single cell experiments [[30], [31], [32]].

As a software implementation to these ends, we present Seidr, a software toolkit that enables researchers to create community networks, perform backboning and perform a variety of other tasks related to gene networks. Our toolkit is efficient and follows evidence-based well performing workflows. Currently, Seidr performs inference using thirteen distinct methods, but a high-level implementation based around multi-processing (OpenMP), message parsing interface (MPI), and the Armadillo linear algebra framework makes the inclusion of new methods in a parallel, shared-memory architecture a straightforward effort. Further, the modular design allows users to import networks calculated with external tools into Seidr and add them to communities, or post-process them in one of many ways.

We used Seidr to reaffirm that community networks and dynamic edge pruning (backboning) both perform better than single methods, congruent with Marbach et al. and Coscia and Neffke [4,5]. We show that community networks are less biased and perform very well compared to any single method in sensitivity, specificity and precision. Precision and recall are especially important in imbalanced problems such as gene regulatory networks, where true negatives far outweigh true positive labels. In addition to network aggregation, we also show that edge pruning in general, and backboning specifically, improved the characteristics of the generated networks.

To illustrate a number of applications, we used Seidr to infer a network based on a real-world study of drought response in *Picea abies*. In addition, we calculated another network for a compendium of unstressed needles.

Firstly, we calculated centrality statistics of all nodes in the drought and unstressed networks. The drought network showed more relevant associations between centrality and GO terms in our GSEA analysis. In addition, genes with putative involvement in drought stress were significantly associated with centrality values in the drought-specific network only, highlighting the importance of creating experiment specific networks.

In order to further gather context about local relationships of genes we partitioned the graph using InfoMap, which resulted in 16 distinct modules. We chose parameters that would result in more modules so as to cluster smaller, more specific groups of genes even if the expression profiles between modules were similar. Of the three modules which were enriched for stress-related GO terms (modules 2, 5 and 6), module 2 was of particular interest as the expression profile corresponded well to the abscisic acid measurements in Haas et al. [23]. Additionally, we identified six highly central transcription factors within that module, which present high interest targets for future genetic modification experiments.

Another application of gene networks is the inference of possible gene function of non-annotated genes. Functional analysis of the neighborhood of non-annotated genes revealed three diverse candidates, which were selected due to their high centrality values in the drought network. The first candidate MA_10432675g0010, is annotated as a cellulase. Genes in its neighborhood are similarly enzymes involved in cell wall modification. Sasidharan, Voesenek, and Pierik [33] review various mechanisms of cell wall reorganization as a response to stresses, suggesting MA_10432675g0010 acts also in response to abiotic stresses to reorganize cell wall structure. The second candidate, MA_69177g0010, has no domain annotation. Only one of its neighbours has been annotated as a mitogen-activated kinase (MAPK). Sinha et al. [34] discuss the role of MAPKs in plants in cellular signaling as a response to abiotic stress, which would place MA_69177g0010 as part of this signaling chain. Finally, MA_10192193g0010 has a neighbour annotated as a photolyase and another as CONSTANS-like. Photolyases are known to deal with DNA-repair in response to light damage [35]. Drought stress can lead plants to have reduced ability to manage light and therefore generate reactive oxygen species (ROS), which can lead to DNA damage similar to excess UV-light [36,37]. The other neighbour is annotated to CONSTANS-like 16 (a photoperiod-sensing gene typically associated with flowering), which has recently been shown to lead to chlorophyll accumulation when over-expressed in *Petunia* [37]. Further, a similar gene has recently been shown to improve drought tolerance in sugarcane when over-expressed [38] via maintenance of photosynthesis and by enhancing the antioxidant and osmotic capabilities of the plant. We therefore infer MA_10192193g0010 to play a role in similar processes *i.e.,* ROS detoxification and photosynthesis upkeep.

## 4 Limitations of the study

Benchmarking tools and estimating accuracy is a non-trivial process in systems biology, due to the lack of truly comprehensive gold-standards. Especially in genome-wide studies such as the expression profiling datasets used as input in the present manuscript, gene annotation resources are notoriously incomplete and biased towards heavily studied biological processes in model organisms. In order to address this caveat and ensure that our results are as reliable as possible to date, we performed the analyses using multiple resources as standards. These resources rely on different underlying set of evidence, which may or not impact the true accuracy calculation. For example, using BioGRID, which is based on protein-protein interactions, to evaluate co-expression relies on multiple assumptions (*e.g.*, guilt by association) ignoring processes that affects it (*e.g.*, post-transcriptional modification, mRNA and protein half-life time, *etc.*). As can be seen in Supplementary Figure 1, 3 and 5, the BioGRID edges with a “genetic” evidence have lower (hence better) edge weight rank then their “physical” evidence counterpart. This is expected, as “genetic” evidence relates more directly to gene co-expression than “physical” protein-protein interaction evidence.

These resources are however the best standards we have to date, and we are confident that any bias they introduce will be similar across datasets and that as such will not affect the comparisons performed in the present manuscript, still we refrained from discussing absolute effects, rather focusing on relative (ratios) effects.

Another consideration in interpreting the results presented in this manuscript, and in any expression-based network in general, is the sample size of the underlying datasets that were used to infer the network. As inference methods rely on the guilt-by-association paradigm by which genes with similar pattern of expression are involved in similar biological processes, the number of samples, as well as the biological spectrum they cover (*e.g.*, growth conditions, mutations, perturbations, tissues, *etc.*) directly affects the relevance of the obtained inference. In our experience, network built from datasets with too few samples (n < 30) or a too narrow spectrum (n < 5), are no more informative than faster weighted correlation (*e.g.*, WGCNA [8]) or fuzzy (*e.g.*, Mfuzz [52]) clustering approaches.

## Author contributions

BS, ND and NRS conceptualized all analyses and selected appropriate datasets for the experiments. BS wrote the majority of the software, performed benchmarking and centrality analyses, created all figures and wrote the manuscript. EZ performed clustering, enrichment and transcription factor analyses as well as curated the list of genes putatively involved in drought stress. AS contributed source code and optimizations to Seidr. BS and ND developed the inference pipeline. BS, ND and NRS edited the manuscript.

## Methods

### Expression profiling data

For *A. thaliana*, 1227 accessions were collected from the NCBI SRA and quantified against the TAIR10 assembly using salmon [39] (v0.13.1) with default options. For *S. cerevisiae* 2129 accessions from the SRA were quantified using salmon (v0.11.2) against the EnsEMBL (r93) assembly. For *D. melanogaster*, 1316 accessions were quantified using salmon/0.11.2 against the FlyBase 6.25 assembly. Each dataset was filtered to have at least 75% aligned reads, resulting in 1216, 1399 and 859 accessions post-filtering respectively. For *P. abies*, only samples from needles were used. The drought network was inferred using raw data (n = 30 samples) from Haas et al. [23], whereas the unstressed network (n = 246 samples) was inferred from a compendium of diverse experiments [43,44] as well as from a diurnal (unpublished) and a seasonal time-series [51].

The count data was imported into R [40] using the tximport package [41], transcript counts were summarized to genes, and the variance stabilizing transformation was applied using DESeq2 [42]. Genes with constant expression (zero variance) were removed. Finally, the median expression of all genes in a sample was subtracted from each gene in that sample, median-centering it. Detailed lists of all accessions can be found in supplementary data S1.

## Network inference

All network inference was done using seidr/0.13.1, with default options unless otherwise specified. In the el-ensemble, llr-ensemble, tigress, pearson, plsnet, and pcor subprograms, the “–scale” option was used to transform data to z-scores prior to inference. The networks were aggregated using the inverse rank product method.

For benchmarking Seidr, the *A. thaliana, D. melanogaster*, and *S. cerevisiae* aggregated networks were further processed as follows: each network backbone was then calculated (for all individual and the community networks) and the edges were filtered for all values of δ in range [0.1,0.2,...,3.5], creating 35 increasingly stringent subsets of the network. Hard cutoffs were then used to create another 35 networks that match the backboned ones in density, *i.e.*, have the same number of edges. Finally, only networks with at least 0.1% edge density were considered for benchmarking. Briefly, edge density is the fraction of edges in a network compared to all theoretical edges, where all genes are connected to all genes. As we only allow one edge between any two genes, this is the triangular matrix without a diagonal: $\frac{N_{genes}\left(N_{genes}-1\right)}{2}$. An edge density of 0.1% equates 22,167 edges in *S. cerevisiae*, 94,222 in *D. melanogaster*, and 485,862 in *A. thaliana*.

For the drought stress analysis, the Norway spruce networks were processed as follows: the aggregated networks were pruned at δ=1.28 (approx. *P*-value of 0.1) using the backboning method. The resulting graph was partitioned using InfoMap [24] version 1.2.1 with parameters:

InfoMap --clu --ftree –N 1 -M 50 --prefer-modular-solution.

Finally, we calculated centrality metrics for nodes via PageRank [45], Strength (weighted degree), Eigenvector centrality, Laplacian centrality [46], Betweenness centrality and Closeness centrality using the default settings in seidr/0.14. In order to summarize all centrality values to a single value per node, we ranked nodes within each algorithm and calculated the median per-node rank across algorithms.

### Statistical analysis

Unless otherwise noted, all statistical analysis was performed in R (version 4.0.2). Gene set enrichment analysis was performed using the fgsea package (version 1.16.0) [47]. All GO enrichment was performed using in-house software implementing the parent child adjusted test from Grossmann et al. [48], while MapMan enrichment uses Fisher's exact test. Both use all non-singleton nodes in the network as the test background (*i.e.*, as the population to compare the set against).

## Gold standards

All gold standard datasets were retrieved in February 2020 from their respective sources (BioGRID [18], KEGG [19], YEASTRACT [20]). For BioGRID, subsets of the full dataset were created based on the evidence type noted for each interaction. For KEGG, the pathway annotations were taken from the R “AnnotationDbi” package [49]. A positive edge in the KEGG standard was defined by two genes sharing at least one pathway, a negative edge between two genes that share no pathways. The “seidr roc” subprogram was then used to calculate true positive rate, false positive rate and precision for all networks and standards. For BioGRID and YEASTRACT where there are no true negatives, we labeled any non-positive edge a negative edge. All plots were then created in R using the “ggplot 2” package [50].

## Software and data availability

Seidr is available at https://github.com/bschiffthaler/seidr. Relevant scripts used in this publication are available at https://github.com/bschiffthaler/seidr-manuscript/. The unfiltered network data for all networks in this manuscript can be downloaded from Mendeley Data at https://dx.doi.org/10.17632/gzvyc42k57.2. The actual binary files (in the sf seidr format) of the different networks containing the results of all methods are available upon request, as they are of significant size and unnecessary to reproduce the results presented in this manuscript.

## Declaration of competing interest

The authors declare that they have no known competing financial interests or personal relationships that could have appeared to influence the work reported in this paper.
